# Frailty in older adults and their association with social determinants of Health. The SABE Colombia Study

**DOI:** 10.25100/cm.v50i2.4121

**Published:** 2019-06-30

**Authors:** José M. Ocampo-Chaparro, Carlos A. Reyes-Ortiz, Ximena Castro-Flórez, Fernando Gómez

**Affiliations:** 1 Universidad del Valle, Facultad de Salud, Departamento Medicina Familiar, Cali, Colombia.; 2 Universidad Libre, Facultad Ciencias de la Salud, Departamento de Medicina Interna, Grupo Interinstitucional de Medicina Interna (GIMI 1), Cali, Colombia; 3 The University of Texas at Houston, Department of Internal Medicine McGovern Medical School, Division of Geriatric and Palliative Medicine, Houston Texas, USA; 4 Florida Agricultural & Mechanical University, Institute of Public Health, College of Pharmacy and Pharmaceutical Sciences, Tallahassee, Florida, USA; 5 Universidad del Valle, Facultad de Salud, Departamento de Medicina Familiar, Escuela de Medicina, Cali, Colombia.; 6 Pontificia Universidad Javeriana, Departamento de Clínicas Médicas, Cali, Colombia; 7 Universidad de Caldas, Grupo de Investigaciones en Gerontología y Geriatría, Grupo de Epidemiología y Población, Manizales, Colombia

**Keywords:** Frailty, SABE Colombia study, elderly, aging, aged, social determinants of health, socioeconomic factors, health surveys, Colombia, Fragilidad, Estudio SABE Colombia, adultos mayores, determinantes sociales de la salud, vejez, envejecimiento, factores socioeconomicos, encuesta en salud, Colombia

## Abstract

**Objective:**

To estimate the prevalence of frailty and evaluate the relationship with the social determinants of health in elderly residents in urban and rural areas of Colombia.

**Methods:**

The SABE (Health, Wellbeing, and Aging) Colombia project is a cross-sectional study, carried out in 2014-2015, involving 24,553 men and women aged 60 years and older who live in the community in Colombia. For this analysis, we used data from 4,474 participants included as a subsample with grip strength measurements. The frailty syndrome was diagnosed according to the Fried criteria (weakness, low speed, low physical activity, exhaustion, and weight loss). The independent variables were grouped as (a) biological and genetic flow, (b) lifestyle (adverse conditions in childhood) (c) social networks and community, and (d) socio-economic, cultural and environmental conditions. Multiple logistic and linear regression analyses were used to assess the prognostic value of frailty for the outcomes of interest.

**Results:**

The prevalence of frailty was 17.9%. The factors significantly associated with frailty were older age, being women, living in rural areas, having low education, a greater number of medical conditions, insufficient current income, childhood health problems and a poor economic situation in childhood.

**Conclusion:**

Our results support the need to include frailty prevention programs, to improve the socioeconomic health conditions of infants to avoid future development of frailty.

## Resumen

**Objetivo:**

Estimar la prevalencia de fragilidad y evaluar la relación con los determinantes sociales de la salud en ancianos residentes en áreas urbanas y rurales de Colombia.

**Métodos:**

El proyecto SABE (Salud, Bienestar y Envejecimiento) Colombia es un estudio transversal, llevado a cabo en 2014-2015, que involucró a 24,553 hombres y mujeres de 60 años o más que viven en la comunidad en Colombia. Para este análisis usamos datos de 4,474 participantes incluidos como sub-muestra con medidas de fuerza de agarre. El síndrome de fragilidad fue diagnosticado según los criterios de Fried (debilidad, baja velocidad, baja actividad física, agotamiento y pérdida de peso). Las variables independientes se agruparon así: (a) biológicos y caudal genético, (b) estilo de vida (condiciones adversas en la infancia) (c) redes sociales y comunitarias, y (d) condiciones socioeconómicas, culturales y ambientales. Análisis de regresión logística y lineal múltiples se utilizaron para evaluar el valor pronóstico de la fragilidad para los resultados de interés.

**Resultados:**

La prevalencia de fragilidad fue del 17.9%. Los factores asociados significantemente con la fragilidad fueron edad más avanzada, ser mujeres, vivir en área rural, tener baja educación, un mayor número de condiciones médicas, ingresos actuales insuficientes, problemas de salud infantil y una situación económica pobre en la infancia.

**Conclusión:**

Nuestros resultados soportan la necesidad de incluir dentro de los programas de prevención de fragilidad, el mejorar las condiciones socioeconómicas y de salud de los infantes con el fin de evitar el desarrollo futuro de fragilidad.

**Remark**

**Table:** 

**1)Why was this study done?**
Given that Colombia is a country with a great social inequality and heterogeneity in the population we find it necessary to evaluate the association between frailty in older adults with the social determinants of health
**2) What did the researchers do and find?**
A cross-sectional study, including older adults living in the urban and rural community of Colombia. The frailty syndrome has five components: weakness, low speed, low physical activity, exhaustion, and weight loss. Factors significantly associated with frailty were living in rural areas, having low education, insufficient current income, childhood health problems and a poor economic situation in childhood
**3) What do these findings mean?**
Our results support the importance of taking into account socioeconomic and health conditions during early childhood as factors that influence the presentation of frailty as we age.

## Introduction

Aging is characterized by the progressive loss of physical and cognitive abilities ^1^. This process in humans is complex and varied. Older adults (OA) can remain without major disabilities throughout their lifetime; however, others will have a poor quality of life due to multiple biopsychosocial factors. Maintaining the functional independence of the OA until the end of the life course is one of the main goals of care in Geriatrics.

The aging of society is a frequent phenomenon. The increase in the population of OA in most western countries leads to a greater number of frail people ^2^ ^,^ ^3^. Frail OA have an increased risk to develop adverse health outcomes, to get sick, in the decline in functional decline, a greater probability of falls, hospitalization, institutionalization, and death ^4^. In recent years, interest in frailty research has increased. The main reason for this is based on the fact that early detection can prevent or delay the adverse outcomes of frailty ^5^.

Frailty is a clinical-biological syndrome with a pathophysiological basis where the involvement of multiple interrelated body systems occurs, which determines the decrease of both the homeostatic reserve and the ability of the body to adapt and the response to stressors, leading to increased vulnerability, being a predictor of disability and adverse health events ^6^. It is a syndrome that differs from the concepts of disability and comorbidity, but it can be presented simultaneously, as evidenced in a study of cardiovascular health ^7^.

For the approach to frailty, there are currently different theoretical models, the best known is that proposed by Fried *et al*. ^7^, which establishes frailty as a phenotype based on five predefined criteria of physical frailty, widely recognized and frequently used by researchers. Also, the frailty index model proposed by Rockwood *et al.* ^8^, which focuses on frailty such as the accumulation of deficits at different levels through a set of clinical conditions and diseases. The last models assume that multiple domains (social, psychological, physical) are related to the concept of frailty, and are evaluated by a group of questions related to each domain, such as the Tilburg frailty indicator ^9^. and the Gröningen frailty indicator ^10^.

The SABE Colombia study (Health, Welfare, and Aging in OA in Colombia), is the largest health survey in the OA population in Latin America and the Caribbean that was conducted in Colombia between 2014-2015 ^11^. The objective of the study was to obtain information on the health status and social and material conditions of life of the OA, to know their health care needs, social protection and to favor a greater dialogue between research in Public Health and the study of aging. The study was based on the theoretical model of the Social Determinants of Health that suggests the existence of structural determinants, for example, the socio-economic and political context, and intermediate material resources: employment, work, income, housing, environment-that explain the majority of health inequalities ^11^.

The social determinants of health are circumstances in which people live and therefore constitute, in this particular case, circumstances in which people age. These circumstances are determined by differences in the distribution of economic resources and public policies adopted by a country and/or region ^12^ ^-^ ^14^.

In the OA, frailty as a paradigm of modern medicine is understood as a biological entity product of the decrease of the reserve and resistance to external stressors and is the result of cumulative damage in multiple systems, which causes vulnerability and facilitates the appearance of other adverse health outcomes ^6^. Although the biological factors associated with the onset of this geriatric syndrome explain the expected outcome, social determinants in health may be associated with the development of frailty in OA.

The associations of socio-economic conditions of children with physical capacity and frailty vary according to the context of different studies, including geographical location and time of birth (e.g., wars, economic transitions, political changes of government), so association may be more important in some literature reviews than others ^15^ ^-^ ^17^.

Studies in North America, Europe, and South America have focused on economic associations to describe the association between adversities in the transition of life with physical health outcomes in later life ^7^ ^,^ ^15^ ^,^ ^18^. Given that Colombia is the third country with the greatest social inequality, in which we find heterogeneity in the population ^19^, we find it necessary to evaluate the association between frailty in OA with the social determinants of health.

## Materials and methods

### Study population

The SABE COLOMBIA project is a cross-sectional study, carried out between 2014-2015, which involved 24,553 men and women aged 60 years or older living in the urban and rural community of Colombia. For this analysis we use data from 4,474 participants included as a subsample with grip strength measures. The methods and procedures were based on those used in the SABE international study to achieve comparability. The rationale and detailed methodology of the SABE Colombia has been described in another document ^20^.

### Measurement of the social determinants of health

In accordance with the multilevel theoretical model of the health determinants proposed in this article, the variables were divided according to the social determinants of Health, starting with the proximal ones (level one and two) and as the table progresses Vertical distribution placing the most distal (level three to five) ^12^.

#### First level: Biological factors and genetic flow

These are the factors of the individual that affect their health and therefore are not modifiable. For this level, the variables were included: age, gender, and race.

#### Second level: Lifestyle factors of the individual

These are variables that represent the different habits of life and personal behaviors. For this level, the variables were included: the economic situation of poverty in childhood or having gone hungry, presence of chronic diseases. We consider this last variable at this level due to the fact that chronic non-communicable diseases have an important lifestyle component in their etiology.

#### Third level: Social and Community Network Factors

They are all social and community influences, being able to influence the behaviors and habits of life of individuals. For this level, the variables were included: Marital status, witnessing violence in childhood and origin

#### Fourth level: socioeconomic, cultural and environmental conditions factors. 

For this level, the variables were included: education, economic income, adversity in health, social affiliation and access for recent problems to health services.

### Measurement of physical frailty

For the measurement of frailty, we use the operational definition of the frailty phenotype developed by Fried *et al* ^7^. The five original features retained in this analysis included "involuntary weight loss," "self-reported exhaustion," "low walking speed," "weakness," and "low physical activity" with some modifications. 

Unintentional weight loss was defined as the self-reported loss of 10 pounds (3 kg) during the previous three months. The weight was measured with a SECA precision scale and the height with a stadiometer on a wall without a base. Fatigue/exhaustion was defined as a negative answer to the following question: "Do you have a lot of energy (yes / no)"; or a positive response to `` Have you ruled out many of your activities or interests (yes / no), '' based on two questions about the Geriatric Depression Scale (scale used in SABE to measure depressive symptoms). The slowness was defined as belonging to the lowest quintile (less than 0.8 m/s) in the three-meter speed test (range, 0.1 to 1.96 m/s), adjusted for sex and height of agreement with short physical performance battery standards ^21^. Weakness was measured by assessing grip strength using a hand dynamometer (Jamar Hydraulic Hand Dynamometer^®^). For participants able to take the test, weakness was defined according to gender and body mass index (BMI) ^22^. For men, the BMI was grouped into four categories: <22.9; 23.0-25.5; 25.6-28.1 and> 28. For each category, the cut-off points for grip strength were set at <19; <22; <22; and <23 respectively. For women, BMI was classified as <24.4; 24.5-27.4; 27.5-30.8; and> 30.8. The corresponding grip strength cut-off points were <11; <12; <14 and <14. And finally, low physical activity was measured using a form adapted to the scale of advanced activities of Reuben's daily life ^23^. Thus, it was defined as a negative response to: "Walk, at least three times a week, between 9 and 20 blocks (1.6 km) without resting?" As previously recommended, 7 participants were classified as frail (presence of 3 or more components), pre-frail (1-2 components) or non-frail (0 component).

### Statistical analysis

A descriptive analysis was carried out on the breakdown of the variables of the social determinants of health, frailty components and other characteristics of the study by sex. The differences were tested by the Chi-square and the T-test. Chi-square and ANOVA tests were performed to test the differences by frailty categories in the social conditions of the life cycle and other characteristics of the study. Two multiple regression models were estimated. The first model included frailty as a dependent categorical variable with frail subjects (code = 1) compared to pre-frail and non-frail subjects (code = 0), using logistic regression models to obtain odds ratios (OR) and 95 confidence intervals. % (CI) to identify variables associated with frailty. The second model included frailty as the dependent continuous variable - [which has a normal distribution-] from 0 to 5, using linear regression models to obtain non-standardized beta coefficients (B) with standard errors (ER) and standardized beta coefficients (β) to identify variables associated with frailty. The statistical level of significance was established at *p* <0.05. Data were analyzed using SAS version 9.4 (SAS Institute, Cary, North Carolina, USA).

## Results

The sample size is 4,474 older Colombians, over 60 years, residents in Colombian territory distributed in 32 departments and 4 cities that are grouped in 5 regions and 16 sub-regions, participants of the National Health, Welfare and Aging survey, SABE Colombia. The events of interest presented and the measurements made for the population of OA were taken taking into account estimates of events of interest with an estimated prevalence of at least 3%. There is sample size for the country with proportional allocation for the urban and rural strata that participate in the results presented, controlling the effect of allocation by forced inclusion.

Table 1 shows the distribution of social factors, health conditions and components of the frailty phenotype and differences between men and women.

**Table 1:** Distribution of social factors, health, and components of the frailty phenotype and differences by sex of the SABE Colombia study, 2015

	Total n= 4,474	Men n= 1,862	Women n= 2,612	
Characteristics	Mean± SD, or n (%)	Mean± SD, or n (%)	Mean± SD, or n (%)	*p*-value
**First Level**				
Age	69.3 ± 6.9	69.6 ± 7.1	69.0 ± 6.9	0.0085
**Race**				
Indigenous	315 (7.0)	170 (9.1)	145 (5.5)	<0.0001
Black	295 (6.6)	138 (7.4)	157 (6.0)	
Mulatto	129 (2.9)	64 (3.4)	65 (2.5)	
White	1422 (31.8)	545 (29.3)	877 (33.6)	
Mestizo	1920 (42.9)	808 (43.4)	1112 (42.6)	
Other	393 (8.8)	137 (7.4)	256 (9.8)	
**Second Level**				
Childhood economic situation of poverty or famine.	1549 (34.6)	708 (38.0)	841 (32.2)	<0.0001
Chronic diseases (≥2)	1724 (38.5)	542 (29.1)	1182 (45.2)	<0.0001
**Third Level**				
Marital status: single	1954 (43.7)	505 (27.1)	1449 (55.5)	<0.0001
Witness of intrafamily physical violence	854 (19.1)	339 (18.2)	515 (19.7)	0.2051
**Origin**				
City	3520 (78.7)	1387 (74.5)	2133 (81.6)	<0.0001
Town	266 (5.9)	128 (6.9)	138 (5.3)	
Rural	688 (15.4)	347 (18.6)	341 (13.1)	
**Fourth Level**				
Insufficient income	3336 (74.6)	1432 (76.9)	1904 (72.9)	0.0024
**Scholarity (years)**				
0	823 (18.4)	353 (18.9)	470 (18.0)	0.0999
1-5	2618 (58.5)	1063 (57.1)	1555 (59.5)	
6-11	798 (17.8)	332 (17.8)	466 (17.9)	
12+	235 (5.3)	114 (6.2)	121 (4.6)	
**Childhood health adversity**			
Poor health or stay in bed >1 month	918 (20.5)	389 (20.9)	529 (20.2)	0.6020
**Type of insurance affiliation**				
Contributory	2011 (44.9)	806 (43.3)	1205 (46.1)	0.0021
Subsidized	2290 (51.2)	970 (52.1)	1320 (50.6)	
Not affiliated	101 (2.3)	59 (3.2)	42 (1.6)	
Other	72 (1.6)	27 (1.4)	45 (1.7)	
**Access due to recent health problems**				
Yes	1069 (23.9)	386 (20.7)	683 (26.1)	<0.0001
No	97 (2.2)	54 (2.9)	43 (1.7)	
Others (unknown or does not apply)	3308 (73.9)	1422 (76.4)	1886 (72.2)	
**Functional factors**				
BMI* (kg/m^2^)	26.9 ± 4.9	25.7 ± 4.0	27.8 ± 5.3	<0.0001
Walking speed (m/seg)	0.73 ± 0.3	0.79 ± 0.3	0.69 ± 0.2	<0.0001
Grip strength (kg)	21.9 ± 9.1	28.4 ± 8.6	21.9 ± 9.1	<0.0001
Chronic diseases (≥2)	1724 (38.5)	542 (29.1)	1182 (45.2)	<0.0001
**Frailty Phenotype**				
Without frailty (none component).	840 (18.8)	406 (21.8)	434 (16.6)	<0.0001
Pre-frail (1-2 components)	2834 (63.3)	1186 (63.7)	1648 (63.1)	
Frail (presence of 3 or more components)	800 (17.9)	270 (14.5)	530 (20.3)	

* BMI: Body mass index.

% = Percentage.

SD = standard deviation.

Chronic diseases: hypertension, diabetes mellitus, acute myocardial infarction, ischemic cerebrovascular disease, chronic obstructive pulmonary disease, arthritis, and cancer.

*p* values were obtained by the Chi-square and the T-test and indicate differences between men and women.

The population had a mean age of 69.3 ±6.9 years, mostly women (58.4%), 34.6% had a childhood history of a poor socioeconomic situation or had reported hunger in the course of their life, 20.5% had a children's history of health adversities and 19.1% had a childhood history of having witnessed physical violence. Regarding the frailty phenotype, 18.8% were non- frail, 63.3% were pre- frail and 17.9% were frail. Compared to men, women were younger at the time of the survey, mostly living together without a partner and comparatively had less history of poor socioeconomic status. Men had comparatively better economic income compared to women, a lower body mass index and fewer medical conditions that constituted current pathological history, less proportion of current insufficient income, higher BMI and number of medical conditions. Women had a slower walking speed and lower grip strength and in general they can be considered to be frailer. 

**Figure 1:**
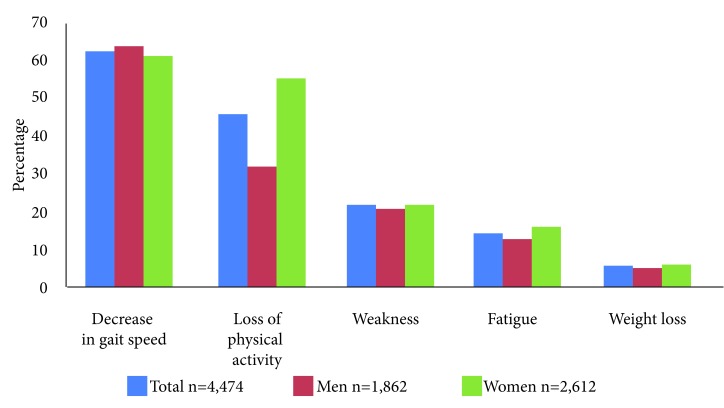
Prevalence of frailty phenotype components. * Differences between men and women, *p* values obtained with the Chi-square test.

**Figure 2:**
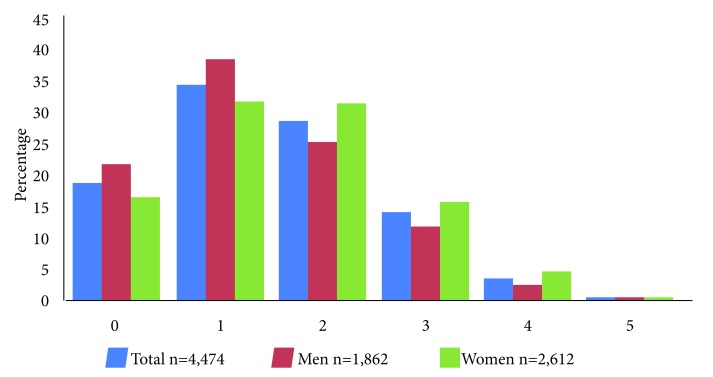
Prevalence of the number of components (from 0 to 5) of the frailty phenotype. * *p*-value obtained with the Chi-square test indicates a global difference between men and women through the components from 0 to 5.

Figure 1 shows the prevalence of frailty phenotype components. The highest percentage is observed for the decrease in gait, and the lowest for weight loss. Women had greater loss of physical activity (*p* <0.001) and fatigue (*p*= 0.008) than men. Figure 2 shows the prevalence of the number of components (from 0 to 5) of the frailty phenotype. Women had a greater number of components (2, 3 and 4) than men (*p* <0.001).

Table 2 shows the bivariate analyses of the characteristics of the study disaggregated by frailty categories. Compared to non-frail and pre-frail people, frail people are older, are women, live without a partner, have more medical conditions, a higher proportion of insufficient income, and a poor economic situation in childhood. It is found that among pre-frail and frail elders comparatively to those who do not have a frail phenotype have fewer years of education and are equally in number, non-frail elders who reach a higher level of schooling compared to pre-frail and frail.

**Table 2:** Bivariate analysis, the study sociodemographic characteristics by disaggregation of the frailty categories, SABE Colombia 2015

	No-Frail, N=840	Pre-Frail N=2,834	Frail N=800	*p*-value
	Mean ± SD, or percentage	Mean ± SD, or percentage	Mean ± SD, or percentage	
**First Level**				
Age	66.6 ± 5.4	69.1 ± 6.7	72.7 ± 7.9	<.0001
**Gender**				
Women	51.7	58.1	66.2	<0.0001
Men	48.3	41.9	33.8	
**Race**				
Indigenous	17.8	63.2	19.0	0.0177
Black	18.6	63.4	18.0	
Mulatto	18.6	67.4	14.0	
White	17.7	62.9	19.4	
Mestizo	20.4	63.9	15.7	
Other	15.8	60.8	23.4	
**Second Level**				
**Childhood economic situation of poverty or famine**				
No	18.8	64.4	16.8	0.0229
Yes	18.7	61.3	20.0	
**Chronic medical conditions**				
0-1	21.4	64.9	13.7	<0.0001
≥2	14.7	60.8	24.5	
**Third Level**				
**Marital Status**				
With couple	20.3	64.3	15.4	<0.0001
Without couple	16.8	62.1	21.1	
**Witness of intrafamily physical violence**				
No	18.3	63.7	18.0	0.2622
Yes	20.7	62.1	17.2	
**Origin**				
City	19.8	63.1	17.1	0.0023
Town	14.3	64.3	21.4	
Rural	15.0	64.4	20.6	
**Fourth Level**				
**Education (years)**				
0	15.2	19.0	19.8	<0.0001
1-5	51.1	58.7	65.5	
6-11	24.2	17.4	12.6	
12+	9.5	4.9	2.1	
**Current material circumstances**				
**Income**				
Sufficient	20.6	63.9	15.5	0.0192
Insufficient	18.1	63.2	18.7	
**Childhood health adversity: Poor health or stay in bed >1 month**				
No	19.4	63.5	17.1	0.0063
Si	16.3	62.6	21.0	
**Type of insurance affiliation**				
Contributory	21.6	62.6	15.8	<0.0001
Subsidized	16.0	64.0	20.0	
Not affiliated	22.2	63.9	13.9	
Other	23.7	63.4	12.9	
**Access due to recent health problems**				
Yes	14.8	60.7	24.5	<0.0001
No	19.6	59.8	20.6	
Others (unknown or does not apply)	20.0	64.3	15.7	

*p*-values were obtained by Chi-square and ANOVA test

Table 3 shows multiple regression analyses to predict frailty. In Model 1, using logistic regression, there are significantly higher probability reasons for being frail in people with the following characteristics: being a woman, having a low education, a greater number of medical conditions, having current insufficient incomes and a poor economic situation in childhood, many of these situations converge only in the gender situation because as we saw earlier, being a woman implies adverse social situations. In model 2, using linear regression, a significantly higher score is found to present frailty in older age, to be women, to have low education, a greater number of medical conditions, insufficient income, child health problems and a poor economic situation in childhood.

**Table 3:** Logistic and linear multiple regression of frailty prediction: SABE Colombia 2015

	Model 1, logistic regression		Modelo 2, lineal regression	
	OR (CI 95%)	*p*-value	*B* (SE), β	*p*-value
**First Level**				
Age	1.08 (1.07-1.09)	<0.0001	0.04 (0.00) 0.27	<0.0001
Women (vs Men)	1.47 (1.22-1.76)	<0.0001	0.22 (0.03) 0.10	<0.0001
**Race**				
Indigenous	0.99 (0.71-1.37)	0.9316	0.01 (0.06) 0.004	0.7994
Black	0.87 (0.61-1.23)	0.4254	-0.07 (0.06) -0.02	0.2933
Mulatto	0.71 (0.42-1.23)	0.2224	-0.08 (0.09) -0.01	0.3836
White (reference)	1.00		0.00	
Mestizo	0.82 (0.68-0.99)	0.0377	-0.06 (0.03) -0.03	0.0651
Other	1.22 (0.92-1.63)	0.1655	0.08 (0.06) 0.02	0.1346
**Second Level**				
Childhood economic situation of poverty or famine (yes vs. no)	1.28 (1.08-1.53)	0.0049	0.06 (0.03) 0.03	0.0516
Chronic medical conditions ≥2 (vs 0-1)	1.72 (1.45-2.03)	<0.0001	0.25 (0.03) 0.11	<.0001
**Third Level**				
No partner (vs having a partner)	1.12 (0.94-1.33)	0.2000	0.04 (0.03) 0.02	0.2161
Witness of intrafamily physical violence (yes vs. no)	0.97 (0.79-1.21)	0.8114	-0.04 (0.03) -0.01	0.3134
**Origin**				
City (reference)	1.00		0.00	
Town	1.45 (1.05-2.02)	0.0257	0.15 (0.06) 0.03	0.0190
Rural	1.36 (1.08-1.71)	0.0092	0.18 (0.04) 0.06	<0.0001
**Fourth Level**				
**Education (years)**				
0	1.93 (1.11-3.34)	0.0194	0.27 (0.07) 0.10	0.0003
1-5	2.16 (1.28-3.66)	0.0041	0.31 (0.07) 0.14	<0.0001
6-11	1.66 (0.96-2.89)	0.0722	0.15 (0.07) 0.05	0.0438
12+ (reference)	1.00			
**Current material circumstances**				
Insufficient income (vs. sufficient)	1.24 (1.02-1.51)	0.0298	0.09 (0.03) 0.04	0.0090
Childhood health adversity: Poor health or stay in bed >1 month (yes vs. no)	1.17 (0.97-1.43)	0.1067	0.08 (0.04) 0.03	0.0393
**Type of insurance affiliation**				
Contributory (reference)	1.00		0.00	
Subsidized	1.22 (1.01-1.45)	0.0334	0.10 (0.03) 0.05	0.0016
Not affiliated	0.89 (0.44-1.80)	0.7407	-0.02 (0.12) -0.002	0.8833
Other	1.14 (0.61-2.12)	0.6769	0.02 (0.10) 0.003	0.8313
**Access due to recent health problems**				
Yes (reference)	1.00		0.00	
No	1.02 (0.59-1.74)	0.9540	0.07 (0.10) 0.01	0.4836
Other (unknown or does not apply)	0.59 (0.49-0.71)	<0.0001	-0.21 (0.03) -0.08	<0.0001

OR = odds ratios;

CI = Confidence interval;

B = non-standardized beta coefficient;

SE = Standard Error;

β = standardized beta coefficient. Model 1: The dependent variable is frail (code = 1) vs non- frail or pre- frail (code = 0); Model 2: the dependent variable is frailty, total score (0-5)

## Discussion

The objective of this study was to describe the association between the social determinants of Health with the three stages of frailty proposed by Fried ^7^, in the OA sub-sample that participated in the SABE COLOMBIA project carried out in 2014 and 2015.

This study allowed identifying the prevalence and social determinants of health consistently associated with frailty in Colombian OA. 17.9% of Colombian OA were frail and frailty was associated with older age, being a woman, being single, with low educational level, insufficient income and having comorbidities (>2 chronic conditions). In addition, in the OA, having experienced a poor economic situation or having suffered hunger in early childhood and health adversities or having been in bed for more than a month during childhood, were more likely to be frail.

Regarding the prevalence of frailty we found (17.9%), it is consistent with the prevalence reported in Latin America and the Caribbean, ranging from 7.7% to 42.6% ^22^. Thus, the concept of 1 in 5 Latin American older adults is corroborated as frail ^22^. In rural areas in Colombia, a slightly lower prevalence was found at 12.2% ^24^, and in an urban older adult population in Cali, the prevalence was also slightly lower than ours ^25^. The prevalence of pre-frail found (63.3%) is consistent with previous studies using the same criteria in similar populations ^26^, which show that early identification in pre-frail and frail OA could have implications for prognosis, optimize care and plan interventions.

Despite considerable research on frailty, there is controversy today about the nature, definition, prevalence, and characteristics of OA in the stages of frailty ^27^, for example, the three traditional explanatory models are known as they are the phenotype, the accumulation of deficits and that of multiple domains ^7^ ^-^ ^10^. However, the psychological and social factors in relation to the stages of frailty have not been widely studied; our research allows us to know consistently that the multilevel theoretical model of social determinants of health has the capacity to discriminate the three stages of frailty proposed by Fried domains ^7^. These findings are useful for health professionals, researchers, and decision-makers in public health.

The levels of social and psychological functioning can play an important role in the development of frailty. For example, in the first level of social determinants of health, the three variables that have been most frequently included in studies of risk factors to develop frailty are age, sex, and education ^28^. When these related factors are analyzed, age has been constantly associated with the development of frailty, especially in advanced ages, suggesting that frailty is a progressive, more significant condition after 80 years ^29^. Women are usually frailer than men, probably because they live longer and as in this study they have a greater number of comorbidities ^22^.

In addition, women have a greater loss of muscle mass during aging and are more likely to develop sarcopenia, a key factor in frailty ^29^. The finding of the ethnic relationship, in our case indigenous, with frailty has not been reported. Previously, the association of blacks with the development of frailty has been reported ^29^ ^,^ ^30^. In previous research on frailty in African Americans of the cardiovascular health study (CHS) a 4-fold probability was found in black non-obese women than in white women ^31^. In turn, in a recent longitudinal systematic review regarding protective or risk factors to develop frailty, age and ethnicity were found to be important sociodemographic factors for the development of frailty ^28^. Studies have shown how the black race, like the indigenous people in this study, is an important indicator of low socioeconomic status and is associated with poor health and high risk of mortality ^29^.

In the second level, we found the association between frailty and adverse conditions in childhood (evidenced by a poor economic situation or having suffered hunger and health adversities or having been in bed for more than a month) which has been previously reported ^15^. Alvarado, in the SABE international study, found that adverse conditions in childhood (hunger, poor health, and poor socio-economic conditions), conditions in adulthood (low educational level) and current (insufficient income) were associated with frailty when aging in both men as in women ^15^. This considerably reinforces the role of socioeconomic factors throughout life as a fundamental element in the development of frailty when aging ^32^.

The number of comorbidities and within them cardiovascular diseases, included in those evaluated in this study, is another factor related to frailty ^33^. In most studies done in Latin America, the number of comorbidities is closely related to frailty ^15^ ^,^ ^24^,^34^. A recent study shows how chronic conditions through the association between frailty and physical abuse in childhood and psychological violence by the couple in adulthood ^35^.

For the third level, we evaluate the association between frailty with marital status, origin, and witness of violence. The finding of marital status, in our case, those who have no partner, and frailty has been a constant in Latin American countries, ^22^ ^,^ ^36^ while on the other hand, the protective effect of having a partner for the development of frailty ^37^.

Rural origin as a risk factor for the development of frailty has been previously reported ^24^. The sociodemographic and economic characteristics evidenced in rural areas are likely the reason for this association ^24^. Thus, in a study of Colombian rural elders, it was found that advanced age, female sex, the presence of comorbidity, dependence on activities of daily living, depressive symptoms, cognitive impairment and poor self-perception of health were factors associated with the development of frailty ^24^.

Violence in childhood has been closely related to alterations in functional capacity as we age ^22^ as well with the development of frailty ^38^. Witnessing violence has been associated with depression in women and alcoholism in men as they age ^39^. Also, it has been reported that physical abuse in childhood leaves marks on the trajectory of life, which leads to adverse consequences on health and frailty in aging ^38^, violence and psychological stress ^40^.

On the fourth level, we found an association between frailty with SES conditions such as education, income, health adversities, social affiliation and access to health services. The role of poor socio-economic conditions for the development of frailty is now clear ^41^ ^,^ ^42^. Several studies have found that frailty is associated with low educational level and insufficient income ^41^ ^,^ ^43^, including patients after myocardial infarction ^44^. Likewise, a low educational level has been considered as a key factor for the development of frailty ^45^. It has been insisted that income and schooling do not act directly on the pathophysiology of frailty, but they interfere with individual lifestyle and quality of life and thus in many factors that vary socioeconomic status including sex and age that can influence frailty process ^29^.

The factors related to social security affiliation and the use of health services have been poorly studied ^28^, and our findings corroborate the importance of their association with frailty. Like us, the negative association between affiliation to a private or health security regime and the development of frailty was previously reported ^46^. As an explanation, the finding of the relationship between frailty and low access to health services among older people of Latin origin has been reported ^47^. The explanation for this relationship is based on the screening and identification of individuals who are at risk or with health maintenance strategies and geriatric treatment for chronic conditions are lower in Hispanics, since as in our study, they have low access to primary care services, and therefore greater risk of developing frailty ^46^.

In summary, the overall frailty phenotype found in this study corresponds to very old and single women with low educational level, with insufficient income, and with comorbidities which have been previously referred to in other populations in developed ^48^, and developing countries ^15^.

Among the strengths is that it is a study of a secondary database analysis of the SABE Colombia project, a population survey based on the theoretical model of social determinants of health and that allowed explaining the condition of frailty in Colombian OA. The criteria of Fried et al. were used to assess frailty, which allows us to compare our results with other studies.

One of the limitations was that the population of our study was restricted only to the sub-sample; however, the methodology of probabilistic sampling used in the original survey allowed to ensure representativeness ^20^. As it is a cross-sectional study, it is not possible to determine the direction of the causal association; therefore, longitudinal studies are required to obtain greater strength in the direction of the associations according to the levels of the social determinants of health that explain the development and negative consequences of frailty.

The approach to frailty through the multilevel approach of the social determinants of health becomes a priority that will allow the health professional to identify and treat OA efficiently in their lifetime. In public health, the decision-maker will carry out population interventions to prevent the development, progression and adverse outcomes related to the condition of frailty.

## Conclusion

Our findings provide information on the prevalence of frailty and its main associated factors in Colombian older adults. Also, this study puts a life course approach into perspective when doing the frailty analysis. Our results support the importance of taking into account socioeconomic and health situations during early childhood as factors that influence the presentation of frailty as they age. These data support the need to include frailty prevention programs, not only for older adults with the frailty phenotype but also to improve the socioeconomic health conditions of infants to avoid the development of frailty in the future.
