# Supplementary material for: Frailty in older adults and their association with social determinants of Health. The SABE Colombia Study
Source: Colomb Med (Cali). 2019 Jun 30;50(2):89–101. doi: 10.25100/cm.v50i2.4121 (PMC6774581; doi:10.25100/cm.v50i2.4121)
Supplement: Translation

# Translation: Fragilidad en personas adultas mayores y su asociación con determinantes sociales de la Salud. Estudio SABE Colombia

**Contribución del estudio**

**Table:** 

**1) Por que se realizó este estudio?**
Dado que Colombia es un país con una gran desigualdad social y heterogeneidad en la población, consideramos necesario evaluar la asociación entre la fragilidad en adultos mayores con los determinantes sociales de la salud
**2) Cuales fueron los resultados relevantes del estudio?**
Un estudio transversal, incluyendo adultos mayores que viven en la comunidad urbana y rural de Colombia. El síndrome de fragilidad tiene cinco componentes: debilidad, baja velocidad, baja actividad física, agotamiento y pérdida de peso. Los factores significativamente asociados con la fragilidad fueron vivir en áreas rurales, tener poca educación, ingresos corrientes insuficientes, problemas de salud infantil y una situación económica deficiente en la infancia
**3) Que aportan estos resultados?**
Nuestros resultados apoyan la importancia de tener en cuenta situaciones socioeconómicas y de salud durante la temprana infancia como factores que influyen en la presentación de fragilidad al envejecer

## Introducción

El envejecimiento se caracteriza por la pérdida progresiva de capacidades físicas y cognitivas ^1^. Este proceso en el ser humano es complejo y variado. Las personas adultas mayores (PAM) pueden mantenerse sin discapacidades importantes durante todo su curso de vida; sin embargo, otros tendrán una mala calidad de vida debido a múltiples factores biopsicosociales. Mantener la independencia funcional de las PAM hasta el final del curso de vida es uno de los principales objetivos del cuidado en la Geriatría. 

El envejecimiento de la sociedad es un fenómeno frecuente. El incremento de la población de PAM en la mayoría de los países occidentales conlleva un mayor número de personas frágiles ^2^^,^^3^. Las PAM frágiles presentan un mayor riesgo para desarrollar desenlaces adversos en la salud, para enfermar, en la disminución funcional, mayor probabilidad de caídas, de hospitalización, de institucionalización y de muerte ^4^. En los últimos años el interés por la investigación en fragilidad se ha incrementado. La principal razón para esto se fundamenta por que la detección temprana puede prevenir o retrasar los desenlaces adversos de la fragilidad ^5^.

La fragilidad es un síndrome clínico-biológico con base fisiopatológica donde se produce la afectación de múltiples sistemas corporales interrelacionados, lo que determina la disminución tanto de la reserva homeostática como de la capacidad de adaptación del organismo y la respuesta a los estresores, llevando a un incremento de la vulnerabilidad, siendo predictor de discapacidad y de eventos adversos en salud ^6^. Es un síndrome que se diferencia de los conceptos de discapacidad y comorbilidad, pero se puede presentar de forma simultánea a ellas, como se evidenció en un estudio de salud cardiovascular ^7^.

Para el abordaje de la fragilidad existen diferentes modelos teóricos.Los más conocidos son el propuesto por Fried et al. ^7^, que establecieron la fragilidad como un fenotipo basado en cinco criterios predefinidos de fragilidad física, ampliamente reconocidos y empleados con frecuencia por los investigadores. El modelo del índice de fragilidad propuesto por Rockwood et al ^8^. que enfoca a la fragilidad como el acumulo de déficits a diferentes niveles mediante un conjunto de condiciones clínicas y enfermedades. El último modelo asume que múltiples dominios (social, psicológico, físico) están relacionados con el concepto de la fragilidad, se evalúa mediante un grupo de preguntas relacionadas con cada dominio, como el indicador de fragilidad de Tilburg ^9^ y el indicador de fragilidad de Gröningen ^10^.

El estudio SABE COLOMBIA (Salud, Bienestar y Envejecimiento en PAM en Colombia), es la mayor encuesta de salud en población PAM en Latinoamericana y del Caribe que fue realizado en Colombia entre 2014-2015 ^11^. El objetivo del estudio fue obtener información sobre el estado de salud y las condiciones sociales y materiales de vida de las PAM, conocer sus necesidades de atención en salud, protección social y favorecer un mayor diálogo entre la investigación en Salud Pública y el estudio del envejecimiento. El estudio se fundamentó en el modelo teórico de los Determinantes Sociales de la Salud que sugiere la existencia de determinantes estructurales, por ejemplo, el contexto socioeconómico y político, e recursos materiales intermedios: empleo, trabajo, ingresos, vivienda, entorno-que explican la mayoría de las desigualdades en salud ^11^.

Los determinantes sociales de la salud, son circunstancias en las que viven las personas y por ello constituyen, para este caso en particular, en circunstancias en las que envejecen las personas. Estas circunstancias están determinadas por las diferencias en la distribución de recursos económicos y las políticas públicas adoptadas por un país y/o región ^12^^-^^14^.

En las PAM, la fragilidad como paradigma de la medicina moderna es entendido como una entidad biológica producto de la disminución de la reserva y de resistencia a los estresores externos, y es resultado de un daño acumulativo en múltiples sistemas, que causa vulnerabilidad y facilita la aparición de otros resultados adversos en la salud ^6^. Si bien los factores biológicos asociados con la aparición de este síndrome geriátrico explican el desenlace esperado, los determinantes sociales en salud pueden estar asociados con el desarrollo de fragilidad en las PAM.

Las asociaciones de condiciones socioeconómica de la infancia con la capacidad física con la fragilidad varían según el contexto de diferentes estudios lo que incluye la ubicación geográfica y el momento de nacimiento (ej: guerras, transiciones económicas, cambios políticos de gobierno)^15^^-^^17^.

Los estudios realizados en Norteamérica, Europa y América del Sur se han centrado en las asociaciones económicas para describir la asociación entre las adversidades en la transición del curso de la vida con los resultados de salud física en la vida posterior ^7^^,^^15^^,^^18^. Dado que Colombia es el tercer país con mayor inequidad social, en la que encontramos heterogeneidad en la población ^19^, encontramos necesario evaluar la asociación entre Fragilidad en PAM con los determinantes sociales de la Salud. 

## Materiales y Métodos

### Población de estudio

El proyecto SABE COLOMBIA es un estudio transversal, llevado a cabo entre 2014-2015, que involucró a 24,553 hombres y mujeres de 60 años o más que viven en la comunidad de áreas urbanas y rurales de Colombia. Para este análisis usamos datos de 4,474 participantes incluidos como submuestra con medidas de fuerza de agarre. Los métodos y procedimientos se basaron en los utilizados en el estudio internacional SABE para alcanzar la comparabilidad. El fundamento y la metodología detallada de SABE Colombia se han descrito en otro documento ^20^.

### Medición de los determinantes sociales de la Salud

De acuerdo con el modelo teórico multinivel de los determinantes de la salud propuesto en el presente artículo, se dividieron las variables de acuerdo con los determinantes sociales de la Salud, iniciando por los proximales (nivel uno y dos) y conforme va avanzando la tabla en distribución vertical ubicando los más distales (nivel tres al cinco) ^12^.

#### Primer nivel: factores biológicos y caudal genético

Son los factores del individuo que afectan su salud y que por lo tanto no son modificables. Para este nivel se incluyeron las variables: edad, género y raza.

#### Segundo nivel: factores de estilo de vida del individuo 

Son variables que representan los diferentes hábitos de vida y los comportamientos personales. Para este nivel se incluyeron las variables: situación económica de pobreza en la infancia o haber pasado hambre, presencia de enfermedad crónicas. Esta última variable la consideramos en este nivel por motivo que las enfermedades crónicas no transmisibles tienen un componente de estilos de vida importante en su etiología.

#### Tercer nivel: factores de redes sociales y comunitarias

Son todas las influencias sociales y de la comunidad, siendo capaces de influir en los comportamientos y hábitos de vida de los individuos. Para este nivel se incluyeron las variables: estado Civil, ser testigo de violencia en la infancia y procedencia

#### Cuarto nivel: factores de condiciones socioeconómicas, culturales y ambientales

Para este nivel se incluyeron las variables: educación, ingreso económico, adversidad en salud, afiliación social y acceso por problemas recientes a servicios de salud.

### Medición de fragilidad física

Para la medición de la fragilidad utilizamos la definición operativa de fenotipo de fragilidad desarrollada por Fried y colaboradores ^7^. Las cinco características originales conservadas en este análisis incluyeron "pérdida de peso involuntaria", "agotamiento auto informado", "velocidad de marcha baja", "debilidad" y "baja actividad física" con algunas modificaciones. La **pérdida de peso involuntaria** se definió como la pérdida auto informada de 10 libras (3 kg) durante los tres meses previos. El peso se midió con una escala de precisión SECA y la altura con un estadiómetro en una pared sin zócalo. La **fatiga / agotamiento** se definió como una respuesta negativa a la siguiente pregunta: '' ¿Tiene mucha energía (sí / no) ''; o una respuesta positiva a '' ¿Ha descartado muchas de sus actividades o intereses (sí / no) '', basándose en dos preguntas sobre la Escala de Depresión Geriátrica (escala utilizada en SABE para medir síntomas depresivos). La **lentitud** se definió como perteneciente al quintil más bajo (menos de 0,8 m / seg) en la prueba de velocidad de tres metros (rango, 0,1 a 1,96 m / s), ajustado por sexo y altura de acuerdo con los estándares de la batería de rendimiento físico corto ^21^. La **debilidad** se midió mediante la evaluación de la fuerza de agarre utilizando un dinamómetro de mano (Jamar Hydraulic Hand Dynamometer®). Para los participantes capaces de tomar la prueba, la debilidad se definió de acuerdo con el género y el índice de masa corporal (IMC) ^22^. Para los hombres, el IMC se agrupó en cuatro categorías: <22.9; 23.0-25.5; 25.6-28.1 y> 28. Para cada categoría, los puntos de corte para la fuerza de agarre se establecieron en <19; <22; <22; y <23 respectivamente. Para las mujeres, el IMC se clasificó como <24,4; 24,5-27,4; 27.5-30.8; y> 30.8. Los puntos de corte de la fuerza de agarre correspondientes eran <11; <12; <14 y <14. Y finalmente, la **baja actividad física** se midió utilizando una forma adaptada de la escala de actividades avanzadas de la vida cotidiana de Reuben ^23^. Así, se definió como una respuesta negativa a: "Camina, al menos tres veces por semana, entre 9 y 20 cuadras (1.6 Km) sin descansar?" Como se recomendó anteriormente,^7^ los participantes se clasificaron en frágiles (presencia de 3 o más componentes), pre-frágiles (1-2 componentes) o no frágiles (0 componente).

### Análisis estadístico

Se realizó un análisis descriptivo sobre el desglose de las variables de los determinantes sociales de la salud, componentes de fragilidad y otras características del estudio por sexo. Las diferencias fueron probadas por el Chi-cuadrado y el T-test. Se realizaron pruebas de Chi- cuadrado y ANOVA para probar las diferencias por categorías de fragilidad en las condiciones sociales del ciclo de vida y otras características del estudio. Se estimaron dos modelos de regresión múltiple. El primer modelo incluyó la fragilidad como variable categórica dependiente con sujetos frágiles (código= 1) en comparación con sujetos precarios y no débiles (código= 0), utilizando modelos de regresión logística para obtener odds ratios (OR) e intervalos de confianza del 95% (IC) para identificar variables asociadas con la fragilidad. El segundo modelo incluyó la fragilidad como la variable continua dependiente -que tiene una distribución normal- de 0 a 5, usando modelos de regresión lineal para obtener coeficientes beta no-estandarizados *(B*) con errores estándar (ER) y coeficientes beta estandarizados (β) para identificar variables asociadas con la fragilidad. El nivel estadístico de significación se estableció en *p* <0.05. Los datos se analizaron utilizando SAS versión 9.4 (SAS Institute, Cary, Carolina del Norte, EE. UU.).

## Resultados

El tamaño de la muestra es de 4,474 ancianos colombianos, mayores de 60 años, residentes en territorio colombiano distribuidos en 32 departamentos y 4 ciudades que se encuentran agrupadas en 5 regiones y 16 subregiones, participantes de la encuesta nacional de Salud, Bienestar y Envejecimiento, SABE Colombia. Los eventos de interés presentados y las mediciones realizadas para en la población de PAM fueron tomadas teniendo en cuenta estimaciones de eventos de interés con prevalencias estimadas de al menos un 3%. Se tiene un tamaño muestral para el país con asignación proporcional para los estratos urbano y rural que participan en los resultados presentados, controlando el efecto de asignación por inclusión forzosa. 

En la Tabla 1 se muestra la distribución de factores sociales, condiciones en salud y componentes del fenotipo frágil y diferencias entre hombres y mujeres. 

**Tabla 1:** Distribución de factores sociales, en salud y componentes del fenotipo frágil y diferencias por sexo del estudio SABE Colombia, 2015

	Total n=4,474	Hombres n=1,862	Mujeres n=2,612	
Características	Media± DE, ó n (%)	Media± DE, ó n (%)	Media± DE, ó n (%)	Valor *p*
**Primer Nivel**				
Edad	69.3 ± 6.9	69.6 ± 7.1	69.0 ± 6.9	0.0085
**Raza**				
Indígena	315 (7.0)	170 (9.1)	145 (5.5)	<0.0001
Negro	295 (6.6)	138 (7.4)	157 (6.0)	
Mulata	129 (2.9)	64 (3.4)	65 (2.5)	
Blanco	1422 (31.8)	545 (29.3)	877 (33.6)	
Mestizo	1920 (42.9)	808 (43.4)	1112 (42.6)	
Otro	393 (8.8)	137 (7.4)	256 (9.8)	
**Segundo Nivel**				
Situación económica de pobreza o hambruna.	1549 (34.6)	708 (38.0)	841 (32.2)	<0.0001
Enfermedades crónicas (≥2)	1724 (38.5)	542 (29.1)	1182 (45.2)	<0.0001
**Tercer Nivel**				
Estado Civil: Sin pareja	1954 (43.7)	505 (27.1)	1449 (55.5)	<0.0001
Testigo de violencia física intrafamiliar	854 (19.1)	339 (18.2)	515 (19.7)	0.2051
**Procedencia**				
Ciudad	3520 (78.7)	1387 (74.5)	2133 (81.6)	<0.0001
Pueblo	266 (5.9)	128 (6.9)	138 (5.3)	
Rural	688 (15.4)	347 (18.6)	341 (13.1)	
**Cuarto Nivel**				
Ingreso insuficiente	3336 (74.6)	1432 (76.9)	1904 (72.9)	0.0024
**Escolaridad (en años)**				
0	823 (18.4)	353 (18.9)	470 (18.0)	0.0999
1-5	2618 (58.5)	1063 (57.1)	1555 (59.5)	
6-11	798 (17.8)	332 (17.8)	466 (17.9)	
12+	235 (5.3)	114 (6.2)	121 (4.6)	
**Adversidad en Salud**				
Pobre salud o estadía en cama >1 mes	918 (20.5)	389 (20.9)	529 (20.2)	0.6020
**Tipo de afiliación a salud**				
Contributivo	2011 (44.9)	806 (43.3)	1205 (46.1)	0.0021
Subsidiado	2290 (51.2)	970 (52.1)	1320 (50.6)	
No afiliado	101 (2.3)	59 (3.2)	42 (1.6)	
Otro	72 (1.6)	27 (1.4)	45 (1.7)	
**Acceso por problemas recientes en salud**				
Si	1069 (23.9)	386 (20.7)	683 (26.1)	<0.0001
No	97 (2.2)	54 (2.9)	43 (1.7)	
Otros ( desconocido o no aplica)	3308 (73.9)	1422 (76.4)	1886 (72.2)	
**Factores funcionales**				
IMC* (kg/m^2^)	26.9 ± 4.9	25.7 ± 4.0	27.8 ± 5.3	<0.0001
Velocidad de la marcha (m/seg)	0.73 ± 0.3	0.79 ± 0.3	0.69 ± 0.2	<0.0001
Fuerza de agarre (kg)	21.9 ± 9.1	28.4 ± 8.6	21.9 ± 9.1	<0.0001
Enfermedades crónicas (≥2)	1724 (38.5)	542 (29.1)	1182 (45.2)	<0.0001
**Fenotipo de fragilidad**				
Sin fragilidad (ningún componente).	840 (18.8)	406 (21.8)	434 (16.6)	<0.0001
Pre frágil (1-2 componentes)	2834 (63.3)	1186 (63.7)	1648 (63.1)	
Frágil (presencia de 3 ó mas componentes)	800 (17.9)	270 (14.5)	530 (20.3)	

*IMC: Índice de masa corporal.

%= Porcentaje.

DE= desviación estándar.

Enfermedad crónica: Hipertensión, Diabetes Mellitus, Infarto agudo de miocardio, Enfermedad cerebrovascular isquémica, Enfermedad pulmonar obstructiva crónica, artritis y cáncer.

Valores *p* obtenidos por Chi- cuadrado y T-test e indican diferencias entre hombres y mujeres.

La población tenía una edad media de 69.3 ± 6.9 años, en su mayoría mujeres (58.4%), el 34.6% tenía antecedentes infantiles de una mala situación socioeconómica o haber reportado hambre en el transcurso de su vida, el 20.5% tenía antecedentes infantiles de adversidades en salud y el 19.1% tenía antecedentes infantiles de haber presenciado violencia física. En cuanto al fenotipo de fragilidad, el 18.8% fueron no frágiles, el 63.3% fueron pre-frágiles y el 17.9% fueron frágiles. En comparación con los hombres, las mujeres eran más jóvenes al momento de la encuesta, en su mayoría convivían sin pareja y comparativamente tenían menos antecedentes de mala situación socioeconómica. Los hombres presentaban comparativamente mejores ingresos económicos respecto de las mujeres, un menor índice de masa corporal y menos condiciones médicas que constituyeran antecedente patológico actual menos proporción por ingresos insuficientes actuales, mayor IMC y número de condiciones médicas. Las mujeres tenían una velocidad de marcha menor y fuerza de agarre y en general se puede considerar que eran más frágiles.

La Figura 1 presenta la prevalencia de los componentes del fenotipo frágil. Se observa el mayor porcentaje para la disminución de la marcha, y el menor para la pérdida de peso. Las mujeres tenían mayor pérdida de actividad física (*p* <0.001) y fatiga (*p*= 0.008) que los hombres. La Figura 2 presenta la prevalencia del número de componentes (de 0 a 5) del fenotipo frágil. Las mujeres tenían mayor número de componentes (2, 3 y 4) que los hombres (*p* <0.001).

**Figura 1:** Prevalencia de los componentes del fenotipo frágil. *Diferencias entre hombres y mujeres, valores *p* obtenidos con la prueba de Chi-cuadrado.

**Figura 2:** Prevalencia del número de componentes (de 0 a 5)del fenotipo frágil. * Valor *p* obtenido con la prueba de Chi-cuadrado, indica diferencia global entre hombres y mujeres a través de los componentes de 0 a 5.

La Tabla 2, muestra los análisis bivariados de las características desagregadas por categorías de fragilidad. En comparación con las personas no frágiles y pre-frágiles, las personas frágiles tienen mayor edad, son mujeres, viven sin pareja, tienen más condiciones médicas, una mayor proporción de ingresos insuficientes, y una situación económica pobre en la infancia. Se encuentra que entre ancianos pre frágiles y frágiles comparativamente a los que no tiene un fenotipo frágil cuentan con menos años de educación y son igualmente en número, los ancianos no frágiles quienes alcanzan mayor nivel de escolaridad comparado con pre frágiles y frágiles.

**Tabla 2:** Análisis bivariados, estudio de las características sociodemográficas con desagregación de las categorías de fragilidad, SABE Colombia 2015

	No Frágil, N=840	Pre-Frágil N=2,834	Frágil N=800	Valor de p
	Mediana ± DS, ó porcentaje	Mediana ± DS, ó porcentaje	Mediana ± DS, ó porcentaje	
**Primer Nivel**				
Edad	66.6 ± 5.4	69.1 ± 6.7	72.7 ± 7.9	<.0001
**Género**				
Mujer	51.7	58.1	66.2	<0.0001
Hombre	48.3	41.9	33.8	
**Raza**				
Indígena	17.8	63.2	19.0	0.0177
Negro	18.6	63.4	18.0	
Mulato	18.6	67.4	14.0	
Blanco	17.7	62.9	19.4	
Mestizo	20.4	63.9	15.7	
Otro	15.8	60.8	23.4	
**Segundo Nivel**				
**Pobre condición socioeconómica en la infancia o hambruna**				
No	18.8	64.4	16.8	0.0229
Si	18.7	61.3	20.0	
**Condiciones médicas crónicas**				
0-1	21.4	64.9	13.7	<0.0001
≥2	14.7	60.8	24.5	
**Tercer Nivel**				
**Estado Marital**				
Con Pareja	20.3	64.3	15.4	<0.0001
Sin Pareja	16.8	62.1	21.1	
**Presenciar violencia física en la familia**				
No	18.3	63.7	18.0	0.2622
Si	20.7	62.1	17.2	
**Procedencia**				
Ciudad	19.8	63.1	17.1	0.0023
Pueblo	14.3	64.3	21.4	
Rural	15.0	64.4	20.6	
**Cuarto Nivel**				
**Escolaridad (en años)**				
0	15.2	19.0	19.8	<0.0001
1-5	51.1	58.7	65.5	
6-11	24.2	17.4	12.6	
12+	9.5	4.9	2.1	
**Circunstancias materiales actuales**				
**Ingreso**				
Suficiente	20.6	63.9	15.5	0.0192
Insuficiente	18.1	63.2	18.7	
**Adversidad en Salud: pobre salud o haber estado en casa > 1 mes (Infancia)**				
No	19.4	63.5	17.1	0.0063
Si	16.3	62.6	21.0	
**Tipo de afiliación a salud**				
Contributivo	21.6	62.6	15.8	<0.0001
Subsidiado	16.0	64.0	20.0	
No afiliado	22.2	63.9	13.9	
Otro	23.7	63.4	12.9	
**Acceso por problemas recientes en Salud**				
Si	14.8	60.7	24.5	<0.0001
No	19.6	59.8	20.6	
Otros ( desconocido o no aplica)	20.0	64.3	15.7	

Valores *p* obtenidos por Chi- cuadrado y Prueba ANOVA

En la Tabla 3, se muestra análisis de regresión múltiple para predecir fragilidad. En el Modelo 1, usando la regresión logística, existen razones de probabilidad significativamente más altas para ser frágiles en las personas con las siguientes características: ser mujer, tener educación baja, un mayor número de condiciones médicas, tener ingresos insuficientes actuales y una situación económica pobre en la infancia, muchas de estas situaciones confluyen sólo en la situación de género pues como se vió anteriormente, el ser mujer implica situaciones sociales adversas. En el modelo 2, usando la regresión lineal, se encuentra una puntuación significativamente más alta para presentar fragilidad en edad más avanzada, ser mujeres, tener baja educación, un mayor número de condiciones médicas, ingresos insuficientes, problemas de salud infantil y una situación económica pobre en la infancia.

**Tabla 3:** Regresión múltiple logística y lineal de predicción de fragilidad: SABE Colombia 2015

	Modelo 1, Regresión logística		Modelo 2, Regresión lineal	
	OR (IC 95%)	Valor de p	*B* (EE), β	p
**Primer Nivel**				
Edad	1.08 (1.07-1.09)	<0.0001	0.04 (0.00) 0.27	<0.0001
Mujer (vs Hombre)	1.47 (1.22-1.76)	<0.0001	0.22 (0.03) 0.10	<0.0001
**Raza**				
Indígena	0.99 (0.71-1.37)	0.9316	0.01 (0.06) 0.004	0.7994
Negro	0.87 (0.61-1.23)	0.4254	-0.07 (0.06) -0.02	0.2933
Mulato	0.71 (0.42-1.23)	0.2224	-0.08 (0.09) -0.01	0.3836
Blanco (referencia)	1.00		0.00	
Mestizo	0.82 (0.68-0.99)	0.0377	-0.06 (0.03) -0.03	0.0651
Otro	1.22 (0.92-1.63)	0.1655	0.08 (0.06) 0.02	0.1346
**Segundo Nivel**				
Situación económica de pobreza en la infancia o haber pasado hambre (si vs. no)	1.28 (1.08-1.53)	0.0049	0.06 (0.03) 0.03	0.0516
Condiciones médicas crónicas ≥2 (vs 0-1)	1.72 (1.45-2.03)	<0.0001	0.25 (0.03) 0.11	<.0001
**Tercer Nivel**				
Sin compañero (vs tener compañero)	1.12 (0.94-1.33)	0.2000	0.04 (0.03) 0.02	0.2161
Testigo de violencia física en la familia (si vs. no)	0.97 (0.79-1.21)	0.8114	-0.04 (0.03) -0.01	0.3134
**Procedencia**				
Ciudad (referencia)	1.00		0.00	
Pueblo	1.45 (1.05-2.02)	0.0257	0.15 (0.06) 0.03	0.0190
Rural	1.36 (1.08-1.71)	0.0092	0.18 (0.04) 0.06	<0.0001
**Cuarto Nivel**				
**Educación (años)**				
0	1.93 (1.11-3.34)	0.0194	0.27 (0.07) 0.10	0.0003
1-5	2.16 (1.28-3.66)	0.0041	0.31 (0.07) 0.14	<0.0001
6-11	1.66 (0.96-2.89)	0.0722	0.15 (0.07) 0.05	0.0438
12+ (referencia)	1.00			
**Circunstancias materiales actuales**				
Ingreso Insuficiente (vs. suficiente)	1.24 (1.02-1.51)	0.0298	0.09 (0.03) 0.04	0.0090
Adversidad en Salud: pobre salud o estar en cama > 1 mes (si vs. no)	1.17 (0.97-1.43)	0.1067	0.08 (0.04) 0.03	0.0393
**Tipo de afiliación a salud**				
Contributivo (referencia)	1.00		0.00	
Subsidiado	1.22 (1.01-1.45)	0.0334	0.10 (0.03) 0.05	0.0016
No afiliado	0.89 (0.44-1.80)	0.7407	-0.02 (0.12) -0.002	0.8833
Otro	1.14 (0.61-2.12)	0.6769	0.02 (0.10) 0.003	0.8313
**Acceso por problemas de salud recientes**				
Si (referencia)	1.00		0.00	
No	1.02 (0.59-1.74)	0.9540	0.07 (0.10) 0.01	0.4836
Otro (desconocido no aplica)	0.59 (0.49-0.71)	<0.0001	-0.21 (0.03) -0.08	<0.0001

OR=odds ratios;

IC=Intervalo de confianza;

*B* = coeficiente beta no-estandarizado;

EE= Error Estándar;

β = coeficiente beta estandarizado.

Modelo 1: La variable dependiente es fragilidad (código=1) vs No frágil o Pre-Frágil (código=0); Modelo 2: la variable dependiente es fragilidad, puntaje total (0-5)

## Discusión 

El objetivo de este estudio fue describir la asociación entre los determinantes sociales de la Salud con las tres etapas de la fragilidad propuestos por Fried ^7^, en la sub-muestra de PAM que participaron en el proyecto SABE COLOMBIA realizada en los años 2014 y 2015.

Este estudio permitió identificar la prevalencia y los determinantes sociales de la Salud consistentemente asociados con fragilidad en PAM colombianos. El 17.9% de las PAM colombianos eran frágiles y la fragilidad estaba asociada con mayor edad, ser mujer, ser soltero, con bajo nivel educativo, ingresos insuficientes y tener comorbilidades (>2 condiciones crónicas); además, en las PAM el haber experimentado una pobre situación económica o haber sufrido hambre en la temprana infancia y adversidades en salud o haber permanecido en cama por más de un mes durante la infancia, tenían mayor probabilidad de ser frágiles. 

Respecto a la prevalencia de fragilidad encontrada (17.9%) es consistente con la prevalencia reportada en Latinoamérica y el Caribe con un rango entre el 7.7% a 42.6% ^22^. Se corrobora así el concepto de 1 de cada 5 adultos mayores latinoamericanos es frágil ^22^. En áreas rurales en Colombia se encontró una prevalencia ligeramente menor a esta de 12.2%^24^, y en una población adulta mayor urbana en Cali, la prevalencia también fue levemente menor a la nuestra ^25^. La prevalencia de pre-fragilidad hallada (63.3%) es consistente con estudios previos utilizando los mismos criterios en poblaciones similares ^26^, lo cual muestra que la identificación temprana en PAM pre frágiles y frágiles podría tener implicaciones para el pronóstico, optimizar la atención y planificar intervenciones. 

A pesar de una considerable investigación sobre la fragilidad, existe controversia en la actualidad sobre la naturaleza, la definición, la prevalencia y las característico de las PAM en los estadios de la fragilidad ^27^, por ejemplo son conocido los tres modelos explicativos tradicionales como son el fenotipo, el acumulo de déficits y el de múltiples dominios ^7^^-^^10^. Sin embargo, los factores psicológicos y sociales en relación con los estadios de fragilidad no han sido ampliamente estudiados, nuestra investigación permito conocer de manera consistente que el modelo teórico multinivel de los determinantes sociales de la salud tiene capacidad para poder discriminar los tres estadios de fragilidad propuestos por Fried dominios ^7^. Estos hallazgos son de utilidad para los profesionales de la salud, investigadores y tomadores de decisión en salud pública.

Los niveles de funcionamiento social y psicológico pueden tener un papel importante en el desarrollo de la fragilidad. Por ejemplo, en el primer nivel de determinantes sociales de la salud, Las tres variables que con mayor frecuencia han sido incluidas en los estudios de factores de riesgo para desarrollar fragilidad son edad, sexo y educación ^28^. Cuando se analizan estos factores relacionados, la edad ha sido asociada de manera constante con el desarrollo de fragilidad, especialmente en edades avanzadas, sugiriendo que la fragilidad es una condición progresiva, más significativa después de los 80 años ^29^. Las mujeres comúnmente son más frágiles que los hombres, probablemente debido a que ellas viven más tiempo y al igual que en este estudio tienen un mayor número de comorbilidades ^22^.

Además, las mujeres presentan mayor pérdida de masa muscular durante el envejecimiento y son más propensas a desarrollar sarcopenia, un factor clave en fragilidad ^29^. El hallazgo de la relación de etnia, en nuestro caso indígenas, con fragilidad no ha sido reportado. Previamente se ha reportado la asociación de raza negra con el desarrollo de fragilidad ^29^^,^^30^. En una investigación previa sobre fragilidad en afroamericanos del estudio de salud cardiovascular (CHS) se encontró una probabilidad de 4 veces mayor en mujeres negras no obesas que en mujeres blancas ^31^. A su vez, en una revisión sistemática longitudinal reciente respecto a factores protectores o de riesgo para desarrollar fragilidad, se encontró que la edad y la etnia son factores sociodemográficos importantes para el desarrollo de fragilidad ^28^. Los estudios han mostrado como la raza negra, al igual que los indígenas en el estudio, son un importante indicador de bajo nivel socioeconómico y está asociado con salud deficiente y alto riesgo de mortalidad ^29^.

En el segundo nivel encontramos la asociación entre fragilidad y condiciones adversas en la infancia (evidenciados por una pobre situación económica o haber sufrido hambre y adversidades en salud o haber permanecido en cama por más de un mes) ha sido informado previamente ^15^. Alvarado, en el estudio SABE internacional encontró que las condiciones adversas en la infancia (hambre, pobre salud y pobres condiciones socioeconómicas), condiciones en la adultez (bajo nivel educativo) y actual (ingreso insuficiente) se asociaron con fragilidad al envejecer tanto en hombres como en mujeres ^15^. Esto refuerza considerablemente el papel de los factores socioeconómicos a través de la vida como elemento fundamental en el desarrollo de fragilidad cuando se envejece ^32^.

El número de comorbilidades y dentro de ellas las enfermedades cardiovasculares, incluidas dentro de las evaluadas en este estudio, es otro factor relacionado con fragilidad ^33^. En la mayoría de estudios hechos en Latinoamérica, el número de comorbilidades está estrechamente relacionado con fragilidad ^15^^,^^24^^,^^34^. Un estudio reciente, muestra como las condiciones crónicas mediante la asociación entre fragilidad y abuso físico en la infancia y violencia psicológica por la pareja en la edad adulta ^35^.

Para el tercer nivel evaluamos la asociación entre fragilidad con estado civil, procedencia, testigo de violencia. El hallazgo sobre el estado marital, en nuestro caso, los que no tienen pareja, y fragilidad ha sido una constante en países latinoamericanos, ^22^^,^^36^ mientras del otro lado, es claro el efecto protector de tener un compañero para el desarrollo de fragilidad ^37^.

La procedencia rural como factor de riesgo para el desarrollo de fragilidad ha sido previamente informado ^24^. Es probable que las características sociodemográficas y económicas evidenciadas en las zonas rurales sean la razón de esta asociación ^24^. Es así como en un estudio de ancianos rurales colombianos se encontró que la edad avanzada, sexo femenino, la presencia de comorbilidad, dependencia en las Actividades de la Vida Diaria, síntomas depresivos, deterioro cognoscitivo y mala autopercepción de salud fueron factores asociados con el desarrollo de fragilidad ^24^.

La violencia en la infancia ha sido estrechamente relacionada con alteraciones de la capacidad funcional al envejecer ^22^ como con el desarrollo de fragilidad ^38^. Ser testigo de violencia ha sido asociado con depresión en mujeres y alcoholismo en hombres al envejecer ^39^. Además, se ha reportado que el abuso físico en la infancia deja marcas en la trayectoria de vida, que conlleva a consecuencias adversas en salud y fragilidad al envejecer ^38^, violencia y estrés psicológico ^40^.

El cuarto nivel realizamos asociación entre fragilidad con condiciones SES, educación, ingreso, adversidades en salud, afiliación social y acceso a servicios de salud. Es ahora claro el papel de las malas condiciones socioeconómicas para el desarrollo de fragilidad ^41^^,^^42^. En varios estudios se ha encontrado que la fragilidad está asociada con bajo nivel educativo e ingreso insuficiente ^41^^,^^43^, incluyendo pacientes posteriores a infarto de miocardio ^44^. Así mismo un nivel educativo bajo, ha sido considerada como un factor clave para el desarrollo de fragilidad ^45^. Se ha insistido que el ingreso y la escolaridad no actúan directamente en la fisiopatología de la fragilidad, pero interfieren en el estilo de vida individual y la calidad de vida y así en muchos factores que varían el estado socioeconómico incluyendo sexo y edad que pueden influenciar el proceso de fragilidad ^29^.

Los factores relacionados con la afiliación a seguridad social y el uso de los servicios de salud han sido poco estudiados ^28^, y nuestros hallazgos corroboran la importancia de su asociación con fragilidad. Al igual que nosotros, previamente se reportó la asociación negativa entre la afiliación a un régimen de seguridad privada o de salud y el desarrollo de fragilidad ^46^. Se plantea como explicación, el hallazgo de previamente ha sido reportado la relación entre fragilidad y bajo acceso a los servicios de salud entre ancianos de procedencia latina ^47^. La explicación para esta relación se basa en que el tamizaje e identificación de individuos quienes están en riesgo o con estrategias de mantenimiento de la salud y tratamiento geriátrico para condiciones crónicas son menores en hispanos, puesto que al igual que en nuestro estudio, tienen bajo acceso a los servicios de atención primario, y por tanto mayor riesgo de desarrollar fragilidad ^46^.

En resumen, el fenotipo de fragilidad encontrado en este estudio de mujer muy vieja, soltera con bajo nivel educativo, con insuficiente ingreso y con comorbilidades ha sido referido previamente en otras poblaciones en países desarrollados ^48^, y en desarrollo ^15^.

Dentro de las fortalezas se tiene que es un estudio de análisis secundario de bases de datos del proyecto SABE COLOMBIA, encuesta poblacional fundamentada en el modelo teórico de los Determinantes Sociales de la Salud y que permitió explicar la condición de fragilidad en PAM colombianos. Se empleó para la valoración de fragilidad los criterios de Fried y cols., lo cual permite comparar nuestros resultados con otros estudios.

Una de las limitaciones fue que la población de nuestro estudio se restringió solo a la sub-muestra, sin embargo, la metodología del muestreo probabilístico empleado en la encuesta original permitió asegurar la representatividad ^20^. Al ser un estudio trasversal no es posible determinar la dirección de la asociación causal, por lo anterior se requieren de estudios longitudinales para obtener mayor fortaleza en la dirección de las asociaciones según los niveles de los determinantes sociales de la Salud que expliquen el desarrollo y las consecuencias negativas de la fragilidad.

El abordaje de la fragilidad mediante el enfoque multinivel de los determinantes sociales de la Salud se convierte en una prioridad que permitirá al profesional de salud identificar y tratar de manera eficiente a las PAM en su curso de vida. En salud pública al tomador de decisiones llevar a cabo intervenciones poblaciones para prevenir el desarrollo, progresión y desenlaces adversos relacionados con la condición de fragilidad.

## Conclusión

Nuestros hallazgos ofrecen información sobre la prevalencia de fragilidad y de sus principales factores asociados en adultos mayores colombianos. Además este estudio coloca en perspectiva un enfoque de curso de vida al momento de hacer el análisis sobre fragilidad. Nuestros resultados soportan la importancia de tener en cuenta situaciones socioeconómicas y de salud durante la temprana infancia como factores que influyen en la presentación de fragilidad al envejecer. Estos datos soportan la necesidad de incluir dentro de los programas de prevención de fragilidad, no solo a adultos mayores con el fenotipo de fragilidad, sino también mejorar las condiciones socioeconómicas de salud de los infantes con el fin de evitar un futuro el desarrollo de fragilidad.
